# Dementia research in Ireland: What should we prioritise?

**DOI:** 10.12688/hrbopenres.13563.2

**Published:** 2023-11-02

**Authors:** Carol Rogan, Bernadette Rock, Emer Begley, Barry Boland, Kevin Brazil, Unai Diaz-Orueta, Sarah Donnelly, Michael Foley, Tony Foley, Caoimhe Hannigan, Louise Hopper, Fiona Keogh, Brian Lawlor, Iracema Leroi, Cora O'Neill, Laura O'Philbin, Maria Pertl, Dominic Trépel, Seán Kennelly

**Affiliations:** 1 School of Medicine, Trinity College Dublin, Dublin, D.2, Ireland; 2 The Irish Medical Council, Dublin, D.2, Ireland; 3 The Alzheimer Society of Ireland, Blackrock, Co. Dublin, A94 N8Y0, Ireland; 4 The National Dementia Office (HSE), Tullamore, Co. Offaly, R35 F6F8, Ireland; 5 Department of Pharmacology & Therapeutics, University College Cork, Cork, T12 XF62, Ireland; 6 School of Nursing & Midwifery, Queen's University, Belfast, BT9 7BL, UK; 7 Department of Psychology, Maynooth University, Maynooth, W23 F2H6, Ireland; 8 School of Social Policy, Social Work and Social Justice, University College Dublin, Dublin, Ireland; 9 PPI Ignite Office, Trinity College Dublin, Dublin, D.2, Ireland; 10 Department of General Practice, University College Cork, Cork, T12 XF62, Ireland; 11 Department of Psychology, School of Business, National College of Ireland, Dublin, Dublin 1, Ireland; 12 School of Psychology, Dublin City University, Dublin, D.9, Ireland; 13 Mental Health Ireland, Dunlaoghaire, Co. Dublin, A96 E289, Ireland; 14 Global Brain Health Institute, Trinity College Dublin, Dublin, D.2, Ireland; 15 St James's Hospital, Dublin, D.8, Ireland; 16 Cork Neuroscience Centre, University College Cork, Cork, T12 XF62, Ireland; 17 Division of Population Health Sciences, Royal College of Surgeons in Ireland, Dublin, D.2, Ireland; 18 Department of Age Related Healthcare, Tallaght University Hospital, Dublin, D.24, Ireland; 19 Department of Medical Gerontology, Trinity College Dublin, Dublin, D.2, Ireland

**Keywords:** Dementia, Research Prioritisation

## Abstract

**Background:**

Dementia research prioritisation allows for the systematic allocation of investment in dementia research by governments, funding agencies and the private sector. There is currently a lack of information available in Ireland regarding priority areas for dementia research. To address this gap, a dementia research prioritisation exercise was undertaken, consisting of an online survey of professionals in the dementia field and workshops for people living with dementia and family carers.

**Methods:**

(1) An anonymous online survey of professionals, based on an existing WHO global survey: the global survey was adapted to an Irish context and participants were asked to score 65 thematic research avenues under five criteria; (2) A mixed-methods exercise for people living with dementia and family carers: this involved two facilitated workshops where participants voted on the research themes they felt were important to them and should be addressed through research.

**Results:**

Eight of the top ten research priorities in the survey of professionals ( *n*=108) were focused on the delivery and quality of care and services for people with dementia and carers. Other research avenues ranked in the top ten focused on themes of timely and accurate diagnosis of dementia in primary health-care practices and diversifying therapeutic approaches in clinical trials. Participants in the workshops ( *n*=13) ranked ‘better drugs and treatment for people with dementia’, ‘dementia prevention/ risk reduction’ and ‘care for people with dementia and carers’ as their top priority areas.

**Conclusions:**

Findings from this prioritisation exercise will inform and motivate policymakers, funders and researchers to support and conduct dementia-focused research and ensure that the limited resources made available are spent on research that has the most impact for those who will benefit from and use the results of research.

## Introduction

Prevalence rates for the number of people living with dementia in Ireland range between 0.78 – 1.1% (approximately 100 in 100,000), depending on the international rates used to measure prevalence ( Pierse *et al.*, 2019), with rates expected to more than double by 2045 (Dementia in Europe Yearbook 2019: Estimating the prevalence of dementia in Europe; https://www.alzheimer-europe.org/Publications/Dementia-in-Europe-Yearbooks). Dementia is the only condition amongst the leading causes of death globally without a treatment to prevent, cure or slow its progression although there are hopes that this may change with emerging potential disease modifying therapies such as Lecanemab. Rising dementia prevalence, an ageing population and a lack of disease-modifying treatments represent a significant scientific, medical and socioeconomic challenge for Ireland. Dementia places significant costs on society – both emotional and economic - and these costs represent one of the fastest growing economic burdens on healthcare systems in developed countries. The total direct and informal care costs for dementia are presently estimated at EUR 2 billion per annum in Ireland ( Connolly *et al.*, 2014). Despite this, funding for research in dementia has been disproportionately low when compared with the impact of the disease, and there is a lack of information available in Ireland regarding priority areas for dementia research. It is vital that the limited resources made available are spent on research that has the most impact for those who will benefit from and use the results of research.

The importance of advancing research aimed at reducing the global impact of dementia has been recognised at an international level (e.g. WHO’s Global Action Plan on the Public Health Response to Dementia 2017–2025; European 2nd Joint Action on Dementia, 2016; G7/G8 Dementia Summit, 2013). Considering funding resources for dementia research are limited (despite increases in recent years), it is recommended that research priorities are set at a national and international level, to inform the systematic allocation of investment in dementia research by governments, funding agencies and the private sector ( Shah *et al.*, 2016). This is highlighted in the WHO’s Global Action Plan for Dementia (2017), which states that “successful implementation of research into dementia aligned with identified research priorities and social and technological innovations can increase the likelihood of progress towards better prevention, diagnosis, treatment and care for people with dementia” (p. 32).

On a national level, the Irish National Dementia Strategy (2014) includes a priority area on research and information systems, and there was an initial investment in dementia research at the time of publication. The Health Research Board’s (HRB) programme of applied dementia research, funded by Atlantic Philanthropies and supported by the Department of Health, had strategic relevance to the implementation of the National Dementia Strategy. The aim of this research programme was to support applied research projects in the areas of organisation and delivery of dementia services; management and decision making in dementia care; and social, economic and policy issues in dementia care.

A 2019 report from the HRB and the Health & Safety Executive’s (HSE) National Dementia Office, following their knowledge exchange event, identified many strengths of the dementia research community in Ireland, including: (i) a relatively small research community with good potential for cross-discipline and cross-sector working; (ii) a number of clinical leaders engaged in high quality research; (iii) the establishment of centres for dementia research; (iv) a focal point and coordination role of the HSE’s National Dementia Office and Dementia Research Network Ireland; (v) good potential for coordination between basic, clinical and social research; and (vi) prioritisation of research within the National Dementia Strategy. Ireland now also has a dementia clinical trial network and a strong presence of key sectors such as technology, pharmaceutical, medical devices and diagnostics, which should be harnessed. 

Dementia Research Network Ireland (DRNI) and The Alzheimer Society of Ireland (ASI) are committed to advocating for increased investment in dementia research. To address the gap in information regarding research priorities in Ireland, DRNI and The ASI jointly hosted a Dementia Research Consensus Forum in April 2018. The forum brought together a wide range of stakeholders, representing the views of researchers, practitioners and clinicians, to collectively explore priorities for dementia research. The aim of the forum was to provide a platform to capture feedback from participants in relation to areas of dementia research that should be prioritised. Forty-four participants took part in a research prioritisation exercise, involving groups of eight to nine participants taking part in a facilitated roundtable group discussion. Groups were asked to address three questions: (i) what are the challenges and gaps in dementia research in Ireland?; (ii) what areas of dementia research should be prioritised and why?; and (iii) how can we enhance the relevance and impact of dementia research? Feedback was captured by facilitators at each table and published in an internal report by DRNI and the ASI.

This research forum acted as a first step in reaching consensus on dementia research priorities and the output of the forum was to convene a steering group in Autumn 2018, to further advance dementia research prioritisation. Participants from the original research forum were invited to join the steering group, and fifteen individuals joined. Regular steering group meetings were hosted by DRNI and The ASI between 2018 and 2019.

The steering group discussed various methods that could be used for a formal research prioritisation exercise, including the advantages and disadvantages of each approach. It was agreed that an existing global research prioritisation exercise ( Shah *et al.*, 2016) would be used and tailored where necessary to an Irish context.

The objectives of the Irish research prioritisation exercise were agreed, as follows:

(i) To elicit feedback from relevant professional stakeholders with experience and/or knowledge of dementia/ dementia research on the priority areas for dementia research in Ireland.
(ii) To elicit feedback from people living with dementia and family carers on what areas of dementia research they would like to see prioritised in Ireland.
(iii) To identify the research avenues that professional stakeholders consider to be most important based on their: potential for success; effect on burden reduction; potential for conceptual breakthrough; potential for translation; and equity.
(iv) To provide information to research funders, policymakers and the research community regarding what research should be prioritised, to ensure that the limited resources made available are spent on research that has the most impact for those who will benefit from and use the results of research.

## Methods

The research prioritisation exercise consisted of: (i) an anonymous online survey of professionals (based on the WHO global survey, Shah *et al.*, 2016); and (ii) a mixed-methods exercise for people living with dementia and family carers consisting of facilitated workshops that included research prioritisation exercises, general discussion which was captured, and a ‘money game’.

Sex and gender differences were not taken into consideration in the design of the study. The survey of professionals and the workshops were designed to garner information from participants on prioritisation of dementia research themes, regardless of sex or gender.

### (i) Survey of professionals

To ensure that the global dementia research prioritisation survey of Shah *et al.* (2016) was suitable for an Irish context, steering group members met in three smaller sub-groups, to discuss each thematic research avenue, with each sub-group reviewing 2–3 over-arching themes. The aim of these sub-group meetings was to review the global survey and decide if any thematic research avenues should be added, altered or removed, to suit the Irish context and make the survey more user-friendly and succinct. It was agreed that the Irish survey should remain as faithful as possible to the global survey. Most of the thematic research avenues were unaltered; however, some were re-worded slightly or added to the Irish survey. In addition, some thematic research avenues from the WHO survey were split into two or three separate research avenues in the Irish survey. See Table 1 for details of changes made.

**Table 1.** Additional or Altered Thematic Research Avenues for the Irish Survey.

Research Theme	Thematic Research Avenue in Global Survey	Additional or Altered Thematic Research Avenues for Irish Survey
**Prevention, Identification** ** and Reduction of Risk**	N/A	Understand the influence and interactions of co-morbidities (including sensory impairment) on disease course.
**Diagnosis, Biomarker ** **Development & Disease** ** Monitoring**	N/A	Establish the feasibility and implementation of a dementia register in Ireland.
**Quality of Care for People** ** with Dementia and Their** ** Carers**	Determine the most effective interventions for educating, training and supporting formal and informal carer(s) of people with dementia	Identify models for effectively educating, training & supporting professional carers and health & social care professionals in the field of dementia. ***And*** Identify the range of supports required to most effectively meet the needs of family/unpaid carers of people with dementia.
**Quality of Care for People** ** with Dementia and Their** ** Carers**	Establish norms and standards for the highest quality of care in residential and nursing homes and approaches to assist families of people with dementia to determine the optimal time to consider placement	Develop clinical guidelines and standards for the delivery of dementia care in residential care settings and in the community. ***And*** Identify how to best support people with dementia and their carers in deciding whether and when to enter long-term care.
**Delivery of Care and ** **Services for People with ** **Dementia and Their ** **Carers**	Conduct a comparative cross-national analysis of the intended scope, implementation/ delivery, and outcomes of National Alzheimer Disease and Dementia plans	Evaluate cross-country/cross-system differences and determine their impact on service provision, cost, quality of care and quality of life for the person with dementia ***And*** Identify and evaluate cross-country/cross-system variations in dementia care plans ***And*** Identify the components of the National Dementia Strategy that are most effective at improving outcomes for people with dementia

The final survey, consisting of 65 thematic research avenues, was uploaded to SurveyMonkey®. Steering group members completed the survey on a pilot basis, to ensure that there were no technical issues or formatting errors. This also allowed for feedback on the time required to complete the survey. As a result of the pilot, it was estimated that the survey would take approximately 30–45 minutes to complete.

The anonymous survey collected some basic demographic information including professional role, institution/organisation, geographical location, years of experience working in the dementia field, area of research, personal experience as an informal (unpaid) carer for a person with dementia and gender. This information was collected to ensure there was a good mix of professionals undertaking the survey, a good range of experience in the field amongst participants, as well as a good geographical spread. It also allowed for some comparisons with the global study ( Shah *et al*., 2016). Participants were then provided with a list of 65 thematic research avenues and asked to score each one (rate their level of agreement) under five different criteria:

**(i)**  **potential for success** - is the proposed research likely to be successful in reaching the proposed endpoint within the next decade?;
**(ii)**  **effect on burden reduction** - has this research potential to markedly reduce the burden of dementia?;
**(iii)**  **potential for conceptual breakthrough** - is the research likely to result in a paradigm shift/ be a ‘game changer?’;
**(iv)**  **potential for translation** - is the research likely to lead to practical application, implementation of new knowledge and/or be deliverable at scale?;
**(v)**  **equity** - is the proposed research outcome likely to benefit people as a whole in an equitable/ fair manner?

Participants were asked to choose one answer: “Yes”; “No”; or “Not Sure/I do not know”, for each of the five criteria listed above.

### (ii) Workshops for People living with Dementia and Family Carers

Two facilitated workshops were carried out with people with dementia and family carers in December 2019, in order to explore participants’ views and perspectives on areas of dementia research they feel should be prioritised. The workshops were facilitated by the Research & Development Officer at Trinity College Dublin’s PPI (Public & Patient Involvement) Ignite Programme and members of The ASI and DRNI were in attendance to support the process. The first workshop was held with people with dementia and the second one with family carers, each lasting 1.5 hours. Discussions during the workshops were recorded and subsequently transcribed, with the permission of participants.

The facilitator provided participants with a list of six over-arching research themes, reflecting those contained in the prioritisation survey for professionals, along with a number of thematic research avenues under each theme as follows: (i) Better drugs and treatment for people with dementia (including eight thematic research avenues); (ii) Reducing the risk of developing dementia - How can we affect the risk factors for dementia (including six thematic research avenues)?; (iii) Building public awareness and understanding (including four thematic research avenues); (iv) Care for people with dementia and carers (including twenty thematic research avenues); (v) Effects of dementia on bodies and brains (including twelve thematic research avenues); and (vi) Diagnosing dementia earlier or better (including ten thematic research avenues). Thematic research avenues that were used in the survey for professionals were presented as questions, in order to explain the themes to a public audience. The six over-arching themes were colour-coded to enable clarity and avoid confusion, and each theme was explained by the facilitator. In the carers’ workshop, the themes and questions were discussed as a group, while in the workshop for people with dementia, the various themes and questions were discussed in pairs.

Participants with dementia were supported by staff from The ASI and DRNI, as well as the facilitator, to ensure their understanding of the various themes and questions. Participants were given the opportunity to express their views on the areas of research they felt should be prioritised, and to hear other people’s perspectives on the various themes and questions. Each participant voted on the research questions they felt were important to them and should be addressed through research, by placing a sticker beside relevant questions.

The workshop with carers concluded with a money game. Respondents were each given a hypothetical budget of EUR 5,000 to invest proportionally in different themes. Participants were invited to put the money into one theme or spread it among various themes. This exercise was another means of capturing participants’ views.

### Participants

*(i) Professional Survey*

The population of interest for the survey was relevant professional stakeholders working in the area of dementia/dementia research in Ireland and included clinicians, health and social care professionals, researchers, representatives from government agencies, policymakers and representatives from the community and voluntary sector. A convenience sample ( *n*=150) of members of DRNI and other relevant stakeholders in the field were invited by email to take part in the online survey. Steering group members also sent an invitation email to colleagues within their network who may be interested in taking part in the survey. Details of the survey were posted on DRNI’s and The ASI’s websites and social media accounts (DRNI and The ASI Twitter pages), to allow for additional stakeholders not identified by the steering group to have an opportunity to complete the survey. The aim was to recruit a minimum sample of 50 participants.

*(ii) Facilitated workshops*

Participants were recruited though The ASI’s Dementia Research Advisory Team (DRAT) and Dementia Carers Campaign Network (DCCN). The DRAT consists of a group of people living with dementia and carers/ supporters who are involved in dementia research as co-researchers. These Experts by Experience influence, advise, and work with researchers across Ireland. The Dementia Carers Campaign Network (DCCN) is a group of people who have experience caring for a loved one with dementia. The group aims to be a voice of and for dementia carers in Ireland and to raise awareness of issues affecting families living with dementia. Thirteen people took part in the workshops, consisting of six people living with dementia ( *n*=6) and seven family carers ( *n*=7).

### Ethics approval

Ethics approval for the online survey was obtained from Trinity College Dublin’s School of Medicine Research Ethics Committee. Participants completing the survey provided informed consent for participation through the online survey platform, SurveyMonkey®. Inclusion criteria for participation in the online survey were age >18 years and a stakeholder with professional experience and knowledge of dementia and/ or dementia research.

Ethics approval was not required for the workshops involving people with dementia and carers as these were PPI consultation workshops under normal ASI operations involving the DRAT and DCCN members, with relevant ASI policy and procedures adhered to.

### Analysis

DRNI’s Scientific Project Manager analysed the results of the survey for professionals provided by SurveyMonkey®, using the same scoring methodology as that used by Shah *et al.* (2016). For each thematic research avenue, intermediate scores for each of the five criteria were first calculated by adding the sum of the scores (‘Yes’=1, ‘No’=0 and ‘Not sure/I do not know’=0.5) and dividing by the number of respondents. This resulted in five numbers (one for each of the five scoring criteria) ranging between 0 and 1. Higher scores reflected respondents’ views that a given research avenue would be likely to fulfil a given scoring criterion, for example ‘potential for success’: the proposed research would likely be successful in reaching the proposed endpoint within the next decade; or ‘effect on burden reduction’: the research has potential to markedly reduce the burden of dementia on patients, caregivers and society as a whole; whereas lower scores reflected respondents’ views that a given research avenue would be unlikely to fulfil a given scoring criterion. An overall priority score was then calculated by averaging the five intermediate scores.

In addition, the proportion of ‘Yes’, ‘No’ and ‘Not sure/I do not know’ responses for each research avenue was counted and the frequency of the most common response for each scoring criterion was noted. The mean of the frequencies across the five criteria was calculated to provide an average expert agreement (AEA) score for each research avenue, which complements the overall priority score, providing a measure of cohesiveness or dispersion in the experts’ opinion.

For the facilitated workshops, ASI staff collated the scoring sheets for both workshops. The ASI engaged a professional transcribing service to transcribe the discussions that arose during the workshops.

## Results

### Demographics for survey respondents

One hundred and eight professionals completed the survey ( *n*=108), which is more than twice the number of respondents anticipated. Not all participants completed all parts of the survey, with respondent numbers ranging from 41 to 62 across the themes. Eighty-one (75%) of survey participants identified themselves as researchers, clinicians, or healthcare/ allied healthcare professionals, with seventeen participants (16%) not providing information on their professional role. The remaining participants in the survey ( *n*=10; 9%) identified themselves as policy, patient advocacy, project manager or administration professionals. The majority of respondents (33%) were working in an academic/ research setting, followed by a University Hospital (19%), community healthcare (12%) and a memory clinic (7%). Seventeen respondents (16%) did not identify their type of institution/ organisation. With regard to geographical location, a significant majority of respondents (48%) were based in Leinster, with 12% specifying ‘nationwide’ and 16% providing no response to this question. The remaining 20% of respondents were based in Munster, Connaught and UK & Northern Ireland. Twenty-six participants (24%) sated that they had personal experience as an informal (unpaid) carer for a person with dementia. See Table 2 for details.

**Table 2.** Demographic Details of Survey Respondents.

			Participants ( *N*=108)
**Professional Role**	Healthcare/Allied Healthcare Professional	47	(43.52%)
	Social & Economic Researcher	14	(12.96%)
	Basic & Translational Researcher	10	(9.26%)
	Clinical Researcher	8	(7.41%)
	Policy & Research/ Advocacy	8	(7.41%)
	Project Manager/ Administrator	2	(1.85%)
	Inter -/transdisciplinary Researcher and Independent Researcher/ Consultant	2	(1.85%)
	No Response	17	(15.74%)
**Institution/ Organisaton**	Academic/ Research Institute	36	(33.33%)|
	University Hospital	21	(19.44%)
	Community Healthcare	13	(12.04%)
	Memory Clinic	8	(7.41%)
	Government Department/Body	4	(3.70%)
	Patient/Advocacy Organisation or Charity	4	(3.70%)
	Self Employed	2	(1.85%)
	Carer Organisation	1	(0.93%)
	Community & Hospital	1	(0.93%)
	Clinical Programme	1	(0.93%)
	No Response	17	(15.74%)
**Geographical Location**	Leinster	52	(48.15%)
	Munster	9	(8.33%)
	Connaught	9	(8.33%)
	UK & Northern Ireland	4	(3.70%)
	Nationwide	13	(12.04%)
	No Response	21	(19.44%)
**Years of Experience In Dementia Field**	Early Career (1–10 yrs)	57	(52.78%)
	Mid-Career (11–20 yrs)	26	(24.07%)
	Late Career (21+ yrs)	5	(4.63%)
	No Response	20	(18.52%)
**Personal Experience as an Informal** ** (Unpaid) Carer for a Person With Dementia**	No Yes No Response	65 26 17	(60.19%) (24.07%) (15.74%)
**Gender**	Female	74	(68.52%)
	Male	15	(13.89%)
	Prefer Not to Say/ No Response	19	(17.59%)

### Top 10 research priorities (for professionals)

Eight of the top ten research priorities were focused on the delivery and quality of care and services for people with dementia and carers. Other thematic research avenues ranked in the top ten focused on themes of diversifying therapeutic approaches (e.g. pharmacological and non-pharmacological interventions) for discovery and development in clinical trials; and timely and accurate diagnosis of dementia in primary health-care practices (see Table 3).

**Table 3.** Top 10 research priorities (professionals).

Priority Ranking	Thematic Research Avenue	Research Theme	No of participant responses
1st	Identify models for effectively educating, training & supporting professional carers and health & social care professionals in the field of dementia	Quality of Care for People With Dementia and Their Carers	46
2nd	Identify effective interventions for the psychological and behavioural symptoms associated with dementia	Quality of Care for People With Dementia and Their Carers	46
Joint 3rd	Identify the range of supports required to most effectively meet the needs of family/unpaid carers of people with dementia	Quality of Care for People With Dementia and Their Carers	46
Joint 3rd	Identify the most effective strategies and supports for enabling people with dementia to maintain their interests and abilities for as long as possible	Quality of Care for People With Dementia and Their Carers	46
Joint 5th	Determine how involvement of people with dementia and their carers might be promoted in research prioritisation and funding, and the design and delivery of services	Delivery of Care & Services for People with Dementia and Their Carers	43
Joint 5th	Identify features of adapted care systems (e.g. environment, nursing care, family engagement) for people with dementia during hospital admission, and determine how best to promote their implementation	Delivery of Care & Services for People with Dementia and Their Carers	43
Joint 5th	Diversify therapeutic approaches (e.g. pharmacological and non- pharmacological interventions) for discovery and development in clinical trials for neurodegenerative and other brain diseases that cause dementia	Pharmacological and Non- Pharmacological Clinical-Translational Research	48
Joint 8th	Develop clinical guidelines and standards for the delivery of dementia care in residential care settings and in the community	Quality of Care for People With Dementia and Their Carers	46
Joint 8th	Identify clinical practice and health system-based interventions that would promote a timely and accurate diagnosis of dementia in primary health- care practices	Diagnosis, Biomarker Development & Disease Monitoring	54
Joint 8th	Determine how to address gaps in healthcare for people with dementia, and what may be the quality of life and cost benefits of more effective holistic integrated medical care	Delivery of Care & Services for People with Dementia and Their Carers	43

In relation to the over-arching theme of ‘ **quality of care for people with dementia and carers’**, priority areas were focused on: 1) the effective education, training and support of professional carers and health & social care professionals; 2) effective interventions for non-cognitive symptoms associated with dementia; 3) the supports required to meet the needs of family/ unpaid carers; 4) effective strategies and supports for enabling people with dementia to maintain their interests and abilities for as long as possible; and 5) the development of clinical guidelines and standards for the delivery of dementia care in residential care settings and in the community.

In relation to the over-arching theme of **‘delivery of care and services for people with dementia and carers’**, priority areas were focused on: 1) how to involve people with dementia and their carers in research prioritisation and funding, and the design and delivery of services; 2) identifying features of adapted care systems for people with dementia during hospital admission and determining how best to promote their implementation; and 3) determining how to address gaps in healthcare for people with dementia and investigating the quality of life and cost benefits of a more effective holistic integrated medical care.

### Top 20 research priorities

In addition to the top 10 priorities discussed above, the top 20 research priorities were reviewed. Under the theme ‘delivery of **care & services for people with dementia and their carers’**, priority areas included: 1) management of non-cognitive symptoms of dementia; 2) identifying models of effective end-of-life care; 3) identifying the barriers to equitable access to care; 4) determining optimal case management for people with dementia; and 5) identifying optimal models for organising and delivering care and support to people with dementia and carers across the disease course. Priority areas under the theme of **‘dementia prevention/ risk reduction’** included: 1) understanding the influence and interactions of co-morbidities on disease course; 2) exploring approaches for primary and secondary prevention of dementia; and 3) determining the feasibility, effectiveness, and best way of delivering interventions to alter/modify risk factors for dementia.

In relation to the theme of **‘pharmacological and non-pharmacological clinical-translational research’,** the research avenue in the top twenty priorities was focused on: ‘promoting collaborations to explore more efficient trials, adaptive trials, and combination therapy for dementia. A top twenty priority under the theme of **‘physiology and disease pathogenesis’** was: ‘understanding the contributions of vascular conditions to diseases causing dementia’.

### Top 4 research avenues per over-arching theme

Priority scores for the 65 thematic research avenues in the survey of professionals ranged from 0·57 to 0·89 (see Analysis section for details of how scores were calculated). Higher scores (closer to 1) reflected respondents’ views that a given thematic research avenue would be likely to fulfil a given scoring criterion (e.g. potential for success, effect on burden reduction etc.). See Table 4 for details of the top four thematic research avenues under each over-arching theme, as well as the overall ranking for each thematic research avenue across all seven research themes.

**Table 4.** Top 4 thematic research avenues for each over-arching research theme and overall ranking for each thematic research avenue.

Rank	Thematic Research Avenue	Overall Score (Mean Expert Agreement)	Overall Ranking Across All Themes	No of Participant Responses
Prevention, Identification and Reduction of Risk			
1 ^st^	Understand the influence and interactions of co-morbidities (including sensory impairment) on disease course.	0.84 (71%)	Joint 11th	62
Joint 2 ^nd^	Explore approaches for primary and secondary prevention of dementia, based on known risk and protective factors.	0.81 (69%)	Joint 19th	62
Joint 2 ^nd^	Determine the feasibility, effectiveness, and best way of delivering interventions to alter/ modify risk factors for dementia (e.g. physical activity, diet, cognitive stimulation etc.)	0.81 (69%)	Joint 19th	62
Joint 2nd	Understand the influence and interactions of non-modifiable (e.g. gender, genetics, age) and modifiable (e.g. physical activity, diet and cognitive stimulation) risk and protective factors for dementia in population-based samples.	0.81 (68%)	Joint 19th	62
Public Awareness and Understanding				62
1st	Determine the effectiveness of dementia-inclusive communities that target stigma and discrimination.	0.80 (66%)	Joint 23rd	59
2nd	Determine the feasibility, effectiveness and best way to enhance awareness and understanding, and reduce stigma, of dementia at an individual, community and society level (across all age groups, including school children).	0.79 (66%)	Joint 29th	59
3rd	Determine how knowledge of risk factors for dementia can be effectively translated into public health campaigns messages for dementia prevention.	0.78 (68%)	33rd	59
4th	Understand diversity and cultural differences in attitudes towards people with dementia.	0.70 (58%)	Joint 47th	59
Diagnosis, Biomarker Development & Disease Monitoring				62
1st	Identify clinical practice and health system-based interventions that would promote a timely and accurate diagnosis of dementia in primary health-care practices.	0.85 (74%)	Joint 8th	54
2nd	Determine cultural values and preferences relating to help-seeking for dementia and earlier diagnosis across different settings and stakeholders.	0.79 (64%)	Joint 29th	50
3rd	Clarify the benefit(s) and ethics of early versus late diagnosis for dementia, and if earlier diagnosis is associated with cost benefits.	0.76 (59%)	Joint 34th	49
4th	Establish the feasibility and implementation of a dementia register in Ireland.	0.75 (67%)	Joint 36th	49
Pharmacological and Non-Pharmacological Clinical–Translational Research				62
1 ^st^	Diversify therapeutic approaches (e.g. pharmacological and non-pharmacological interventions) for discovery and development in clinical trials for neurodegenerative and other brain diseases that cause dementia.	0.86 (75%)	Joint 5th	48
2 ^nd^	Promote collaborations to explore more efficient trials, adaptive trials, and combination therapy for dementia.	0.84 (65%)	Joint 11th	48
3rd	Identify, validate, and apply better outcome measures for clinical trials of cognition, function, and other biomarkers for neurodegenerative diseases causing dementia.	0.81 (67%)	Joint 19th	48
4 ^th^	Establish norms/ standardise clinical trial methodology and ethics of conducting research with new pharmacological agents, and non-pharmacologic interventions for diseases causing dementia.	0.71 (51%)	Joint 45th	47
Physiology and Progression of Normal Ageing and Disease Pathogenesis				
1 ^st^	Understand the contributions of vascular conditions to neurodegenerative diseases causing dementia.	0.83 (67%)	Joint 15th	46
2 ^nd^	Understand the role of inflammation and of the immune system in the initiation/onset and progression of neurodegenerative diseases that lead to dementia.	0.79 (63%)	Joint 29th	46
3rd	Investigate how intrinsic biological ageing processes may contribute to the neurodegenerative diseases causing dementia.	0.75 (59%)	Joint 36th	46
4 ^th^	Investigate biological processes of neurodegenerative diseases to understand their contributions to dementia to optimise individualised therapeutic strategies.	0.74 (57%)	Joint 40th	46
Quality of Care for People with Dementia and their Carers				62
1st	Identify models for effectively educating, training & supporting professional carers and health & social care professionals in the field of dementia.	0.89 (83%)	1st	46
2nd	Identify effective interventions for the psychological and behavioural symptoms associated with dementia.	0.88 (80%)	2nd	46
3rd	Identify the range of supports required to most effectively meet the needs of family/ unpaid carers of people with dementia.	0.87 (80%)	Joint 3rd	46
Joint 3rd	Identify the most effective strategies and supports for enabling people with dementia to maintain their interests and abilities for as long as possible.	0.87 (78%)	Joint 3rd	46
Delivery of Care & Services for People with Dementia and Their Carers				
Joint 1st	Determine how involvement of people with dementia and their carers might be promoted in research prioritisation and funding, and the design and delivery of services	0.86 (77%)	Joint 5th	43
Joint 1st	Identify features of adapted care systems (e.g. environment, nursing care, family engagement) for people with dementia during hospital admission, and determine how best to promote their implementation.	0.86 (77%)	Joint 5th	43
3rd	Determine how to address gaps in healthcare for people with dementia, and what may be the quality of life and cost benefits of more effective holistic integrated medical care.	0.85 (72%)	Joint 8th	43
4th	Determine what service and system-level interventions are effective in preventing and managing behavioural symptoms of dementia in long-term care settings.	0.84 (74%)	Joint 11th	43

### Priority ranking by people living with dementia and carers

Participants in the workshops ranked ‘better drugs and treatment for people with dementia’ as their top priority theme, with the following feedback provided by participants around this theme:

*“It’s a question of us all being involved in that kind of global fight. And you know a great result can come from a small country like Ireland.”* (family carer)
*“[A]lot of that kind of collaboration happens (reference to clinical trials) and it could be just that Irish people get a chance to be involved with a drug trial. So it could be starting in Germany but you know that we’re geared up that we say ‘oh we’ve a cohort of people here in Ireland willing to take part’. So that we’re benefiting from that research.”*
  (person living with dementia)

The theme of ‘dementia prevention/ risk reduction’ received the second highest score from workshop participants, with the following feedback provided by participants around this theme:

*“There’s a lot of different risk factors, lots of debate about what’s a risk factor and what’s not, maybe we need more research to learn about what the risk factors are and to tell people ”* (person living with dementia)

Participants referred to the need to engrain messaging about risk factors in young people as they grow up, noting:

“ *I don’t think society is necessarily ready to change their behaviours*.” (person living with dementia)

During a discussion on the theme of ‘building public awareness and understanding’, the following feedback was provided by a person living with dementia:

*“Inclusion and education is probably the best way of reducing [stigma]… giving knowledge to people of what it is, how it affects people in the sense of their dignity, their ability to be included, an inclusion issue.”* (person living with dementia)

On the theme of ‘care for people with dementia and carers’, workshop participants provided the following insights:

“ *the more sign posting and psycho, say educational courses that are done in the early days, if appropriate, … long term has a far bigger impact on the journey for not just the person but with their families*” (person living with dementia)

Participants also emphasised the importance of understanding the person as a whole:

“ *Too often it’s about managing the dementia and not the person… So it’s what are the unmet needs and how can they best be helped… it can be easy to hand out a drug, it can be a little bit more challenging to find out what’s going on*” (person living with dementia)

Concern was expressed regarding the training of professionals in the area of dementia:

“ *I’ve got a lot of concern about training and support when people go into hospital, whether it’s for short term or otherwise … I worry about the standards of care … anybody front line, be it residential, hospitals, medical people, that they would have ongoing up to date mandatory training*.” (person living with dementia)

This reflects findings from the survey of professionals, where ‘educating, training & supporting professional carers and health & social care professionals’ was ranked in number one place.

Under the theme ‘effects of dementia on bodies and brains’, there was a lot of discussion about the need to understand how dementia interacts with other conditions and how different conditions can affect dementia:

“ *I think that there is an interest there about, you know, how does dementia affect your body and how does it affect the cells and … what can we understand about that really, really fine level*.” (person living with dementia)

See Table 5 for results of the priority scoring exercise that took place at the workshops.

Results for the ‘money game’ carried out with carers are detailed in Table 6. The theme that scored the highest in this exercise was ‘care for people with dementia and carers’, followed by ‘better drugs & treatment for people with dementia, ‘public awareness & understanding’ and ‘diagnosing dementia earlier or better’. The remaining themes of ‘reducing the risk of developing dementia’ and ‘effects of dementia on bodies and brains’ were not allocated any money by participants in this exercise.

Table 7 contains details of all results from the survey of professionals, across the seven research themes.

Results for the ‘money game’ carried out with carers are detailed in Table 6. The theme that scored the highest in this exercise was ‘care for people with dementia and carers’, followed by ‘better drugs & treatment for people with dementia, ‘public awareness & understanding’ and ‘diagnosing dementia earlier or better’. The remaining themes of ‘reducing the risk of developing dementia’ and ‘effects of dementia on bodies and brains’ were not allocated any money by participants in this exercise.

**Table 5.** Results of Priority Scoring Exercise at PPI Workshops.

Research Theme	No. of Votes
Better drugs and treatment for people with dementia	31
Reducing the risk of developing dementia - How can we affect the risk factors for dementia?	17
Building public awareness and understanding	14
Care for people with dementia and carers	11
Effects of dementia on bodies and brains	9
Diagnosing dementia earlier or better	7

**Table 6.** Results of Money Game Conducted with Carers.

Research Theme	Money Allocated
Care for people with dementia and carers	EUR 16,000
Better Drugs & Treatment for People with Dementia	EUR 5,000
Public Awareness & Understanding	EUR 2,000
Diagnosing Dementia Earlier or Better	EUR 2,000
Reducing the Risk of Developing Dementia	EUR 0
Effects of Dementia on Bodies and Brains	EUR 0

*Note: A hypothetical budget of EUR35,000 was given to the group of 7 carers*

**Table 7.** Research Priorities By Overall Priority Score.

Rank	Thematic Research Avenue	Over-Arching Research Theme	Overall Priority Score	Average Expert Agreement	No of Participant Responses	Global Survey Ranking ( Shah *et al.*, 2016)
1st	Identify models for effectively educating, training & supporting professional carers and health & social care professionals in the field of dementia.	Quality of Care	0.89	83%	46	Joint 9th
2nd	Identify effective interventions for the psychological and behavioural symptoms associated with dementia.	Quality of Care	0.88	80%	46	Joint 11th
Joint 3 ^rd^	Identify the range of supports required to most effectively meet the needs of family/unpaid carers of people with dementia.	Quality of Care	0.87	80%	46	Joint 7th
Joint 3rd	Identify the most effective strategies and supports for enabling people with dementia to maintain their interests and abilities for as long as possible.	Quality of Care	0.87	78%	46	Joint 7th
Joint 5th	Determine how involvement of people with dementia and their carers might be promoted in research prioritisation and funding, and the design and delivery of services	Delivery of Care & Services for People with Dementia and Their Carers	0.86	77%	43	Joint 51st
Joint 5th	Identify features of adapted care systems (e.g. environment, nursing care, family engagement) for people with dementia during hospital admission, and determine how best to promote their implementation.	Delivery of Care & Services for People with Dementia and Their Carers	0.86	77%	46	Joint 42nd
Joint 5th	Diversify therapeutic approaches (e.g. pharmacological and non- pharmacological interventions) for discovery and development in clinical trials for neurodegenerative and other brain diseases that cause dementia	Pharmacological and Non-Pharmacological Clinical-Translational Research	0.86	75%	48	Joint 3rd
Joint 8 ^th^	Develop clinical guidelines and standards for the delivery of dementia care in residential care settings and in the community.	Quality of Care	0.85	76%	46	Joint 19th
Joint 8th	Identify clinical practice and health system-based interventions that would promote a timely and accurate diagnosis of dementia in primary health- care practices	Diagnosis, Biomarker Development & Disease Monitoring	0.85	74%	54	2nd
Joint 8th	Determine how to address gaps in healthcare for people with dementia, and what may be the quality of life and cost benefits of more effective holistic integrated medical care.	Delivery of Care & Services for People with Dementia and Their Carers	0.85	72%	43	Joint 44th
Joint 11 ^th^	Determine what service and system-level interventions are effective in preventing and managing behavioural symptoms of dementia in long- term care settings	Delivery of Care & Services for People with Dementia and Their Carers	0.84	74%	43	Joint 26th
Joint 11th	Identify models of effective end-of-life care for people with dementia, including advance care planning.	Delivery of Care & Services for People with Dementia and Their Carers	0.84	73%	44	Joint 9th
Joint 11th	Understand the influence and interactions of co-morbidities (including sensory impairment) on disease course.	Prevention, Identification & Reduction of Risk	0.84	71%	62	N/A
Joint 11th	Promote collaborations to explore more efficient trials, adaptive trials, and combination therapy for dementia.	Pharmacological and Non-Pharmacological Clinical-Translational Research	0.84	65%	48	Joint 11th
Joint 15th	Identify the barriers to equitable access to care (e.g. for people from ethnic minorities living with dementia and other hard to reach subgroups) and the service and system level modifications that would help to overcome these factors.	Delivery of Care & Services for People with Dementia and Their Carers	0.83	72%	43	Joint 26th
Joint 15th	Determine how case management for people with dementia should best be structured and delivered, and if it is effective in promoting access to evidence-based interventions and care, and in reducing unnecessary service use and costs	Delivery of Care & Services for People with Dementia and Their Carers	0.83	72%	43	Joint 21st
Joint 15th	Identify optimal models for organising and delivering care and support to people with dementia and carers across the disease course. Evaluate their relative effectiveness.	Delivery of Care & Services for People with Dementia and Their Carers	0.83	70%	44	Joint 7th
Joint 15th	Understand the contributions of vascular conditions to neurodegenerative diseases causing dementia.	Physiology and Progression of Normal Ageing and Disease Pathogenesis	0.83	67%	46	Joint 3rd
Joint 19th	Explore approaches for primary and secondary prevention of dementia, based on known risk and protective factors.	Prevention, Identification & Reduction of Risk	0.81	69%	62	1st
Joint 19th	Determine the feasibility, effectiveness, and best way of delivering interventions to alter/modify risk factors for dementia (e.g. physical activity, diet, cognitive stimulation etc.)	Prevention, Identification & Reduction of Risk	0.81	69%	62	Joint 7th
Joint 19th	Understand the influence and interactions of non-modifiable (e.g. gender, genetics, age) and modifiable (e.g. physical activity, diet and cognitive stimulation) risk and protective factors for dementia in population-based samples.	Prevention, Identification & Reduction of Risk	0.81	68%	62	6th
Joint 19th	Identify, validate, and apply better outcome measures for clinical trials of cognition, function, and other biomarkers for neurodegenerative diseases causing dementia	Pharmacological and Non-Pharmacological Clinical-Translational Research	0.81	67%	48	Joint 17th
Joint 23rd	Identify the most appropriate indicators to measure both the effectiveness and quality of care, across the full spectrum of care settings for people with dementia.	Quality of Care	0.80	69%	45	Joint 35th
Joint 23rd	Identify how to best support people with dementia and their carers in deciding whether and when to enter long-term care.	Quality of Care	0.80	68%	44	Joint 19th
Joint 23rd	Understand the role of assistive and technological devices, including e- health and mobile health technology strategies, for people with dementia and/or their carer(s)	Delivery of Care & Services for People with Dementia and Their Carers	0.80	67%	43	Joint 15th
Joint 23rd	Determine the effectiveness of dementia-inclusive communities that target stigma and discrimination.	Public Awareness & Understanding	0.80	66%	59	Joint 26th
Joint 23rd	Optimise legislation and care systems to promote social participation and protect autonomy of individuals living with dementia	Delivery of Care & Services for People with Dementia and Their Carers	0.80	65%	43	Joint 35th
Joint 23rd	Identify strategies to promote choice across the full spectrum of care settings for people with dementia. Evaluate their effectiveness and cost- effectiveness.	Quality of Care	0.80	64%	45	Joint 57th
Joint 29th	Determine the feasibility, effectiveness and best way to enhance awareness and understanding, and reduce stigma, of dementia at an individual, community and society level (across all age groups, including school children).	Public Awareness & Understanding	0.79	66%	59	59th
Joint 29th	Determine cultural values and preferences relating to help-seeking for dementia and earlier diagnosis across different settings and stakeholders.	Diagnosis, Biomarker Development & Disease Monitoring	0.79	64%	50	Joint 35th
Joint 29th	Understand the role of inflammation and of the immune system in the initiation/onset and progression of neurodegenerative diseases that lead to dementia	Physiology and Progression of Normal Ageing and Disease Pathogenesis	0.79	63%	46	Joint 21st
Joint 29th	Explore methods to detect and reduce abusive behaviour by family, paid caregivers, and health and social care professionals, towards people with dementia.	Delivery of Care & Services for People with Dementia and Their Carers	0.79	63%	43	Joint 35th
33rd	Determine how knowledge of risk factors for dementia can be effectively translated into public health campaigns messages for dementia prevention.	Public Awareness & Understanding	0.78	68%	59	Joint 3rd
Joint 34th	Clarify the benefit(s) and ethics of early versus late diagnosis for dementia, and if earlier diagnosis is associated with cost benefits.	Diagnosis, Biomarker Development & Disease Monitoring	0.76	59%	49	Joint 44th
Joint 34th	Develop improved methods to model the cost of dementia across the disease course, and the relative cost-effectiveness of different interventions and care strategies.	Delivery of Care & Services for People with Dementia and Their Carers	0.76	58%	43	Joint 55th
Joint 36th	Establish the feasibility and implementation of a dementia register in Ireland.	Diagnosis, Biomarker Development & Disease Monitoring	0.75	67%	49	N/A
Joint 36th	Identify the components of the National Dementia Strategy that are most effective at improving outcomes for people with dementia.	Delivery of Care & Services for People with Dementia and Their Carers	0.75	60%	42	N/A
Joint 36th	Investigate how intrinsic biological ageing processes may contribute to the neurodegenerative diseases causing dementia.	Physiology and Progression of Normal Ageing and Disease Pathogenesis	0.75	59%	46	Joint 32nd
Joint 36th	Understand the influence of environmental factors on increasing the risk of dementia and/or progression of the disease.	Prevention, Identification & Reduction of Risk	0.75	55%	62	Joint 21st
Joint 40th	Evaluate cross-country/cross-system differences and determine their impact on service provision, cost, quality of care and quality of life for the person with dementia.	Delivery of Care & Services for People with Dementia and Their Carers	0.74	58%	43	N/A
Joint 40th	Investigate biological processes of neurodegenerative diseases to understand their contributions to dementia to optimise individualised therapeutic strategies	Physiology and Progression of Normal Ageing and Disease Pathogenesis	0.74	57%	46	Joint 21st
Joint 40th	Identify underlying mechanisms of resilience to neurodegenerative diseases causing dementia at all stages (such as cognitive reserve, protective genotypes, and neuroprotection)	Physiology and Progression of Normal Ageing and Disease Pathogenesis	0.74	56%	45	Joint 11th
43rd	Understand the impact of the neurodegenerative diseases causing dementia upon the structure and function of neural systems and networks with the aim of identifying new therapeutic targets.	Physiology and Progression of Normal Ageing and Disease Pathogenesis	0.73	57%	46	Joint 26th
44th	Develop and validate biomarkers—including biological, genetic, behavioural, and cognitive markers—for neurodegenerative brain diseases causing dementia, to identify similarities and differences between diseases and dementia subtypes, and assess progression from pre-clinical (pre-symptomatic) to late-stage diseases.	Diagnosis, Biomarker Development & Disease Monitoring	0.72	57%	54	Joint 15th
Joint 45th	Establish longitudinal cognitive surveillance of healthy individuals to detect earliest changes that distinguish pre-clinical (pre-symptomatic) neurodegenerative diseases causing dementia from normal ageing, and which may be used as endpoints in primary prevention clinical trials	Diagnosis, Biomarker Development & Disease Monitoring	0.71	56%	54	Joint 11th
Joint 45th	Establish norms/standardise clinical trial methodology and ethics of conducting research with new pharmacological agents, and non- pharmacologic interventions for diseases causing dementia.	Pharmacological and Non-Pharmacological Clinical-Translational Research	0.71	51%	47	Joint 35th
Joint 47th	Understand diversity and cultural differences in attitudes towards people with dementia.	Public Awareness & Understanding	0.70	58%	59	Joint 32nd
Joint 47th	Identify the most cost-effective strategies for early identification of dementia.	Diagnosis, Biomarker Development & Disease Monitoring	0.70	52%	54	Joint 17th
Joint 49th	Explore and validate possible drug repurposing (of agents with proven safety and efficacy for the treatment of other outcomes) to find new candidates for dementia trials.	Pharmacological and Non-Pharmacological Clinical-Translational Research	0.69	55%	47	Joint 35th
Joint 49th	Explore and validate strategies to optimise drug delivery into the brain (past the blood-brain barrier/BBB) to prevent or treat dementia.	Pharmacological and Non-Pharmacological Clinical-Translational Research	0.69	54%	48	Joint 26th
Joint 49th	Determine cost-effectiveness of different interventions for dementia risk reduction.	Prevention, Identification & Reduction of Risk	0.69	53%	62	Joint 51st
Joint 49th	Quantify global prevalence and incidence rates of dementia, and survival with dementia, thus clarifying how dementia is evolving in countries worldwide, and whether this relates in predictable ways to changes in known risk determinants.	Diagnosis, Biomarker Development & Disease Monitoring	0.69	51%	49	Joint 46th
Joint 49th	Identify cognitive and functional measures for assessment in pre-clinical (pre-symptomatic) neurodegenerative diseases causing dementia.	Diagnosis, Biomarker Development & Disease Monitoring	0.69	48%	Joint 21st
54th	Identify cross-country/cross-system variations in dementia care resources, their financing, and organisation.	Delivery of Care & Services for People with Dementia and Their Carers	0.68	53%	43	Joint 53rd
55th	Identify and evaluate cross-country/cross-system variations in dementia care plans.	Delivery of Care & Services for People with Dementia and Their Carers	0.67	49%	43	Joint 53rd
Joint 56th	Establish and validate cohorts of highly-defined participants which would be trial-ready to accelerate recruitment for international clinical trials for dementia.	Pharmacological and Non-Pharmacological Clinical-Translational Research	0.66	53%	47	Joint 49th
Joint 56th	Identify, compare, and understand the genetic variability in individuals with different types of dementia, and their potential to explain different progression patterns within and between populations.	Prevention, Identification & Reduction of Risk	0.66	48%	62	Joint 46th
58th	Understand at the basic mechanistic level the gender-related vulnerabilities of neurodegenerative diseases causing dementia.	Physiology and Progression of Normal Ageing and Disease Pathogenesis	0.65	63%	46	Joint 55th
Joint 59th	Understand the basic biological mechanisms of neuronal cell death involved in the initiation/ onset and progression of neurodegenerative diseases causing dementia.	Physiology and Progression of Normal Ageing and Disease Pathogenesis	0.64	61%	46	Joint 32nd
Joint 59th	Understand the basic biological mechanisms of dysfunction of cellular metabolism, and their regulation in the initiation/onset and progression of neurodegenerative diseases that lead to dementia.	Physiology and Progression of Normal Ageing and Disease Pathogenesis	0.64	61%	46	Joint 35th
Joint 59th	Determine the roles of non-neuronal brain cells (such as microglia, astrocytes and macrophages) in pathogenesis and progression of neurodegenerative diseases that cause dementia.	Physiology and Progression of Normal Ageing and Disease Pathogenesis	0.64	59%	46	Joint 46th
62nd	Understand protein misfolding and propagation in the brain and their role in the initiation/ onset and progression of neurodegenerative diseases causing dementia.	Physiology and Progression of Normal Ageing and Disease Pathogenesis	0.63	60%	45	Joint 49th
Joint 63rd	Test pharmacologic and non-pharmacologic interventions in trials in animal models and human studies of pre-clinical (pre-symptomatic) neurodegenerative diseases causing dementia using biomarkers to address pathologic processes	Pharmacological and Non-Pharmacological Clinical-Translational Research	0.60	51%	47	Joint 42nd
Joint 63rd	Determine the feasibility, acceptability, benefits and ethics of screening for pre-clinical (pre-symptomatic) dementia across health systems and cultures.	Diagnosis, Biomarker Development & Disease Monitoring	0.60	48%	50	Joint 26th
65th	Develop and validate animal and other models to capture the pathology and progression of neurodegenerative diseases and accurately represent dementia subtypes.	Physiology and Progression of Normal Ageing and Disease Pathogenesis	0.57	57%	46	Joint 57th

## Discussion

Priority themes for professionals, and those with the lived experience of dementia, relate to the delivery and quality of care and services for people with dementia and carers, dementia prevention/ risk reduction, timely and accurate diagnosis of dementia, clinical–translational research (including both pharmacological & non-pharmacological interventions), understanding physiology & disease pathogenesis, and building public awareness and understanding.

Eight of the top ten research priorities from the survey of professionals were focused on the delivery and quality of care and services for people with dementia and carers. Family carers also prioritised care for people with dementia and carers in the money game, allocating the majority of the hypothetical research funding to this theme. One possible explanation for the survey results relates to the survey itself, where care-related research avenues are likely to score well on the specific weightings (potential for success; effect on burden reduction; potential for conceptual breakthrough; potential for translation; and equity). Another explanation for the prioritisation of care-related research is that it reflects the lack of available treatments, resulting in an emphasis on maximising care to optimise the available benefit for people with dementia and carers. It is interesting to note that workshop participants voted ‘better drugs and treatment’ as their top research priority, again possibly reflecting the current lack of available treatments for dementia.

There were some similarities between the Irish findings and those from the global survey conducted by Shah *et al.* (2016). The global survey found that three of the top ten research priorities were focused on delivery and quality of care for people with dementia and their carers. Another three of the top ten priorities were focused on prevention, identification, and reduction of dementia risk. Diversifying therapeutic approaches in dementia clinical trials was ranked joint 3 ^rd^ in the global survey, 7 ^th^ in the Irish survey and ‘better drugs and treatment’ was the top priority for people with dementia and carers who took part in the facilitated workshops. Another research avenue which was prioritised by both the Irish survey, and the global survey, was around the timely and accurate diagnosis of dementia in primary health-care practices, which was ranked 9th in the Irish survey and 2nd in the global survey.

Shah *et al.* (2016) report that several initiatives carried out in recent years in high-income countries have produced research recommendations for better detection, prevention, and treatment of dementia, and to improve the quality of life of people affected by dementia as well as their families and carers. These are in keeping with Ireland’s recommendations to focus on timely and accurate diagnosis of dementia, dementia prevention (ranked in the top twenty of the survey of professionals and ranked second as a theme for people with the lived experience of dementia) and diversifying therapeutic approaches in clinical trials. In relation to other countries’ focus on improving the quality of life of people affected by dementia as well as their families and carers, this is in keeping with the findings in the Irish survey of professionals, where eight of the top ten research priorities were focused on the delivery and quality of care and services for people with dementia and carers. Whilst it is interesting to compare findings with these other international initiatives, Shah *et al.* (2016) caution that the scope and methods used varied across these various initiatives in other countries, and therefore caution should be exercised when making comparisons.

Being mindful of the different scope and methods used in other initiatives internationally, it is nevertheless of interest to compare findings from a James Lind Alliance Priority Setting Partnership (PSP) exercise carried out by the UK’s Alzheimer’s Society in 2013 ( Kelly *et al.*, 2015). PSPs bring patients, carers and clinicians together to identify and prioritise research questions. Notably, members of the research community are excluded from the process, unless they are a clinician, patient or carer. In line with findings from this Irish study and the global survey ( Shah *et al.* 2016), seven of the top ten research questions in the UK PSP study were on the theme of care (including promoting independence, care in the hospital setting, maintaining nutritional intake, supporting carers, optimal timing for movement into a nursing home and end-of-life care). The other research questions in the top ten of the UK PSP study related to early diagnosis/ effective routes to diagnosis, interventions for non-cognitive symptoms of dementia and design features of dementia friendly environments. Comparing these UK PSP findings to the Irish survey, Irish-based professionals also prioritised the timely and accurate diagnosis of dementia and effective interventions for the non-cognitive symptoms associated with dementia, both of which were ranked in the top ten priorities by professionals. In the Irish survey, research focused on dementia-inclusive communities was ranked 26 ^th^ and therefore wasn’t given as high a priority as the UK PSP study, which ranked this research in their top ten priorities. This difference may reflect the considerable work that has already been undertaken on dementia awareness and dementia-inclusive communities in Ireland in recent years, including the HSE's Understand Together Understand Together campaign ( Galvin *et al.*, 2020).

With regard to the professionals who completed the Irish survey, 75% ( *n*=81) of survey participants identified themselves as researchers, clinicians, or healthcare/ allied healthcare professionals. This is similar to the global survey ( Shah *et al*., 2016), where 88% ( *n*=142) of participants identified themselves as researchers or clinicians. The majority of respondents in the Irish survey (33%) were working in an academic/ research setting, followed by a University Hospital (19%), community healthcare (12%) and a memory clinic (7%). With regard to geographical location, a significant majority of respondents (48%) were based in Leinster, with 20% based in Munster, Connaught or UK & Northern Ireland, 12% specifying their location as ‘nationwide’ and 16% not responding to this question. In the Irish survey, twenty-six participants (24%) stated that they had personal experience as an informal (unpaid) carer for a person with dementia, in comparison to the global survey, where eighteen (11%) of the experts who participated also designated themselves as carers of someone with dementia.

Shah *et al.* (2016) emphasise that the research priorities identified in their global dementia prioritisation exercise will inform and assist the balance of research investment across research themes and quote a Lancet Editorial that states “the quest for new drugs must not overshadow improving today’s care and patients’ lives” ( Lancet, 2014). This is also true for this Irish prioritisation exercise which provides research priorities across seven over-arching themes, spanning basic science, prevention, clinical and social research areas. Workshop participants spoke of the need for researchers to work collaboratively, avoiding duplication or overlapping of research, and the need to build on research:

*“[T]hey should be constantly building on knowledge. Now I’m not saying it’s perfect because we know there is a lot of waste in research. But that should be the idea, that it’s all building and improving and advancing all the time.”* (Family carer)

Participants in the workshops scored ‘better drugs and treatment for people with dementia’ as their top priority theme and participants in the survey of professionals ranked 'research to diversify therapeutic approaches (e.g. pharmacological and non-pharmacological interventions) in clinical trials' in their top ten priorities. Ireland’s first dementia clinical trial network (Dementia Trials Ireland; DTI), funded by the Health Research Board, has recently been established and this will result in an increased focus on intervention studies for prevention and treatment of dementia, including both drug and non-drug approaches across the stages of dementia (i.e. preclinical to advanced stage). 

Workshop participants scored ‘dementia prevention/risk reduction’ as their second highest priority theme and professionals included research avenues under this theme in their top twenty priorities. There has been a greater focus on dementia prevention and the influence of modifiable risk factors, over the last number of years. Our understanding of the potential for dementia prevention has been enhanced through key publications, such as the Lancet Commission reports ( Livingston *et al.*, 2017; Livingston *et al.*, 2020) and the WHO Guidelines ‘Risk Reduction of Cognitive Decline and Dementia’ (2019). It is clear from the thematic research avenues contained in the survey of professionals that there is scope for more research in this rapidly developing area of dementia prevention. 

Although different methodologies were used for the survey of professionals and the workshops for people living with dementia and carers, there was a more narrow spread of scores found in the survey. Looking at the top twenty research priorities ranked by professionals, scores ranged from 0.81 to 0.89 (out of a possible score from 0 to 1). In comparison, there was a wider spread of scores amongst workshop participants when they were voting for their top research priorities, with their first priority (better drugs and treatment) receiving 31 votes, followed by ‘reducing the risk of developing dementia’ which received 17 votes. Similarly, in the ‘money game’ that carers participated in, the majority of funding (EUR 16,000) was allocated to ‘care for people with dementia and carers’, followed by just under a third of that amount (EUR 5,000) for the next theme ‘better drugs and treatments’.

It is interesting to note that workshop participants scored ‘better drugs and treatment’ as their highest priority, however for the ‘money game’ carried out with carers, this was ranked in second place for funding, after ‘care for people with dementia and carers’. The emphasis on care reflects the findings in the survey of professionals, where eight of the top ten research priorities related to care themes. It appears that both themes (‘better drugs & treatments’ and ‘care’) are important for people living with dementia and carers. It would be worthwhile to conduct further workshops with people with dementia and family carers, to investigate priorities amongst these two groups, exploring both commonalities and differences.

Although a EUR35,000 hypothetical budget was given to the seven carers (EUR5,000 each), and all carers used some or all of their budget, only EUR25,000 was allocated to four of the six research themes. An exploration of why some budget remained un-used was not carried out at the workshop although this would be interesting to explore in any future workshops with carers.

Sex or gender analysis of survey participants was not conducted and sex or gender information was not collected from workshop participants. For both the survey and workshop, the main focus was garnering insight from participants, regardless of sex or gender. The absence of sex or gender analysis is not thought to be detrimental to the study.

### Strengths and limitations

Survey respondents consisted of a convenience sample of members of DRNI and other relevant stakeholders in the field who were invited by email to take part in the online survey. Details of the survey were posted on DRNI’s and The ASI’s websites and social media accounts and some respondents may have accessed the survey through these avenues. Although 108 people responded to the survey, it is likely that some people working in the area of dementia/ dementia research in Ireland were not aware of the survey or did not get an opportunity to complete it. It is recommended that the survey is repeated at periodic intervals in order to provide an opportunity for anyone working in the area of dementia/ dementia research to take part.

Given the good balance of professionals who took part in the survey, it is likely that respondent bias has been reduced. However, given that the majority of survey participants (75%) identified themselves as researchers, clinicians, or healthcare/ allied healthcare professionals, it is possible that this group influenced the results of the survey in comparison to the smaller group of policy, patient advocacy, project manager & administration professionals. However, the mean expert agreement was higher than 70% for sixteen of the top twenty priorities, which is a similar finding to the global survey conducted by Shah *et al.*, 2016. The survey is considered to be transparent, systematic, rigorous, replicable and democratic, and limits the effects of personal interests or biases ( Shah *et al.*, 2016). One of the strengths of this prioritisation exercise is that it did not merely ask professionals what areas of dementia research should be prioritised, but rather asked them to consider five important criteria when determining research priorities, as follows: (i) potential for success - is the proposed research likely to be successful in reaching the proposed endpoint within the next decade?; (ii) effect on burden reduction - has this research potential to markedly reduce the burden of dementia?; (iii) potential for conceptual breakthrough - is the research likely to result in a paradigm shift/ be a ‘game changer’?; (iv) potential for translation - is the research likely to lead to practical application, implementation of new knowledge and/or be deliverable at scale?; and (v) equity - is the proposed research outcome likely to benefit people as a whole in an equitable/ fair manner? Another strength of this exercise is the fact that people living with dementia and family carers were involved in determining research priorities, however people at risk of dementia (preclinical and those with mild cognitive impairment) were not included, which is a limitation.

Not all participants completed all parts of the survey, with respondent numbers ranging from 41 to 62 across the themes. It is likely that respondents completed the sections of the survey that reflected their area of research interest and expertise. Respondent numbers decreased as the survey progressed, possibly indicating a level of respondent fatigue. It does not appear that this influenced the ranking of research avenues, as the care-focused themes appeared last in the survey but were ranked the highest by survey respondents. For future prioritisation exercises, it is recommended that the survey is split into four sub-surveys, which could be administered at different timepoints.

Only one research prioritisation workshop took place with people living with dementia, and only one with family carers, resulting in small numbers of PPI representatives ( *n*=13) taking part in this exercise, mainly from Dublin and the surrounding area. Plans to conduct more workshops were curtailed due to the COVID-19 pandemic. When the prioritisation exercise is repeated, more workshops for people living with dementia and family carers should be facilitated, with better geographical spread and including those at risk of dementia.

## Conclusions

For the first time in Ireland, we have insight from professional stakeholders and those with the lived experience of dementia, regarding research avenues that should be prioritised. Findings from this exercise will be valuable to policymakers, funding agencies and the research community, as it informs the systematic allocation of investment in dementia research and reduces the likelihood of research waste.

Research priorities identified through this exercise, include themes around care, prevention/ risk reduction, diagnosis & disease monitoring, clinical-translational research (including both pharmacological & non-pharmacological interventions), understanding physiology & disease pathogenesis, and building public awareness and understanding. These priorities inform and assist the balance of research investment across the research domains of basic science, prevention, clinical and social science. It is vital that collaborative interdisciplinary research is encouraged across the research domains as it is through such interdisciplinary research that we have the best chance to improve outcomes for people with dementia and their families.

Now that research priorities have been identified, it is vital that adequate funding is provided by our national funding agencies, in order to conduct the research. There are a number of crucial factors that heighten the urgency of prioritising research in the area of dementia, including an ageing population, the emotional and economic cost of dementia and the fact that delaying the age of onset, or the progression of dementia, is likely to have significant effects with regard to the enormous associated public health burden ( Ritchie & Ritchie, 2012).

In terms of national policy, DRNI and The ASI will continue to collaborate with the HSE’s National Dementia Office (NDO) to align research priorities from this exercise with priority areas of the National Dementia Strategy and the work programme of the NDO. The NDO can guide the focus, or facilitate meaningful actions, in relation to specific thematic research avenues. DRNI and The ASI will also continue to play an important role in facilitating interdisciplinary research, facilitating recruitment of research participants (through the TeamUp for Dementia Research service operated by The ASI), facilitating transfer and exchange of research knowledge and linking research with policy and practice.

Dementia research priorities will change over time, as new treatments and interventions emerge, advances are made in relation to disease pathology and biomarker development, and research gaps are addressed in relation to care issues. Shah *et al.* (2016) call for WHO Member States, and civil society, to continuously monitor research investments and progress, as well as temporal and geographical trends in dementia incidence, prevalence, and burden. DRNI and The ASI aim to repeat this exercise at periodic intervals in order to provide up-to-date information on dementia research priorities to funders, policymakers and the research community, as well as feeding into international platforms such as the global dementia observatory.

## Data Availability

Trinty’s Access to Research Archive: DRNI Survey Data.xlsx, https://doi.org/10.25546/101186 ( Rogan *et al.*, 2022). Data is held under a Creative Commons Attribution-ShareAlike license (CC-BY-SA). The workshop data is under license by a third party and cannot be shared openly. To request access to data from the facilitated workshops (involving people living with dementia and family carers), contact DRNI: info@dementianetwork.ie or The Alzheimer Society of Ireland: research@alzheimer.ie.
