# Supplementary material for: Dementia research in Ireland: What should we prioritise?
Source: HRB Open Res. 2023 Nov 2;6:12. Originally published 2023 Feb 9. [Version 2] doi: 10.12688/hrbopenres.13563.2 (PMC10638481; doi:10.12688/hrbopenres.13563.2)
Supplement: Peer review history

# Reviewer report: Reviewer response for version 2

**DOI:** 10.21956/hrbopenres.15144.r36905

**Published:** 2023-11-10

**Authors:** Jennifer Bray

**Affiliations:** 1 University of Worcester, Worcester, England, UK

Thank you for taking on board my previous review comments, I hope you agree that the revised article is now stronger as a result. I have a few additional comments, most of which relate to consistency (which was raised in my previous comments). While this may sound picky, it can actually be quite jarring and distracting for the reader, and can potentially cause them to question the quality of your work. I appreciate that as authors you can be too close to the article and not see some of these things, so please take my comments in the constructive way they are intended.

 Participants – I find it odd that you day how many participants there were in the facilitated workshops, but not for the professional survey – instead giving this number in the results section. It’s picky, but for consistency it feels like you should either give the numbers for both activities in the participants section or the results section, rather than one in either.

 Demographics for survey respondents – in the last sentence before Table 2, I think you mean to say ‘(24%) stated’ not ‘(24%) sated’

 General point about consistency – sometimes when using a ‘/’ you have a space after it and sometimes you don’t, e.g. and/or and/ or. I don’t mind which you do, but I think you should be consistent throughout the article and in the tables.

 Table 2 – in the ‘participants’ column there looks like an odd line after one result ‘(33.33%)|’

 You use both ‘health-care’ (e.g. just before table 3) and ‘healthcare’ (2 ^nd^ paragraph after table 3), so again some consistency would be good.

 Top 20 research priorities – part of the first theme is not in bold. You also say ‘top 20’ and ‘top twenty’ which again comes back to consistency. In the 2 ^nd^ paragraph in this section, you’ve got a ‘ before promoting collaborations, but I can’t see a matching ’. I assume it should come after therapy for dementia.

 Priority ranking by people living with dementia and carers – in the second quote the bit saying who it’s by is on a separate line rather than directly after the quote as per the other quotes.

 Table 7 – as this relates to the ranking by professionals, this should be earlier in the article rather than in the section reporting on the priority ranking by people living with dementia and carers. It’s not relevant where it is.

 The paragraph before Table 5 which talks about the money game results is repeated twice. One of the paragraphs can be deleted. In that paragraph, there is no ’ at the end of the ‘better drugs…theme.

 Consistency point – in the final few paragraphs of your discussion section, you start by saying ‘EUR 16,000’ and ‘EUR 5,000’ (i.e. with a space after EUR), but later you say ‘EUR35,000’, ‘EUR5,000’ and ‘EUR25,000’ (i.e. no space after EUR). Please be consistent.

Is the work clearly and accurately presented and does it cite the current literature?

Partly

If applicable, is the statistical analysis and its interpretation appropriate?

Partly

Are all the source data underlying the results available to ensure full reproducibility?

No

Is the study design appropriate and is the work technically sound?

Yes

Are the conclusions drawn adequately supported by the results?

Yes

Are sufficient details of methods and analysis provided to allow replication by others?

Partly

Reviewer Expertise:

Dementia

I confirm that I have read this submission and believe that I have an appropriate level of expertise to confirm that it is of an acceptable scientific standard.

# Reviewer report: Reviewer response for version 2

**DOI:** 10.21956/hrbopenres.15144.r36904

**Published:** 2023-11-02

**Authors:** Francesco Di Lorenzo

**Affiliations:** 1 Hospitalisation and Health Care Santa Lucia Foundation, Rome, Italy

I am happy with authors review

Is the work clearly and accurately presented and does it cite the current literature?

Yes

If applicable, is the statistical analysis and its interpretation appropriate?

Yes

Are all the source data underlying the results available to ensure full reproducibility?

Yes

Is the study design appropriate and is the work technically sound?

Yes

Are the conclusions drawn adequately supported by the results?

Yes

Are sufficient details of methods and analysis provided to allow replication by others?

Yes

Reviewer Expertise:

Dementia

I confirm that I have read this submission and believe that I have an appropriate level of expertise to confirm that it is of an acceptable scientific standard.

# Reviewer report: Reviewer response for version 1

**DOI:** 10.21956/hrbopenres.14809.r34695

**Published:** 2023-07-11

**Authors:** Francesco Di Lorenzo

**Affiliations:** 1 Hospitalisation and Health Care Santa Lucia Foundation, Rome, Italy

This is an interesting report that sheds light on the vision that people working on dementia has on which priorities should be taken in account in Ireland.

 The paper is well written and I just have some comments, which I think their consideration could improve the quality of the paper. 

 Firstly, as a researcher clinician, I think that one of the most important priority is a timely diagnosis. Nowadays, we are in the era of anti-amyloid drugs which have the potential to impact the course of the disease (even if i have to admit the data presented are not very convincing). However, this recent pharmacological advancement, together with other possible treatments, urge the need of a timely diagnosis. We should not forget about that; also because much more clinical research can be performed on patients in order to improve knowledge on the disease. For example, lumbar puncture examination is able to give us insights into the levels of the pathological proteins involved in the pathophysiology of AD (DOI:10.1111/cns.12476 ^1^) and this data can be associated to other clinical means which can help in better stratifying the patient, especially in clinical terms (doi: 10.1159/000090631 ^2^).

 Another issue I would emphasize is the concept of frailty among people with dementia and especially AD (DOI:10.2174/1871527311312040010 ^3^). The identification of these patients would help the clinicians in alleviating their symptoms and reducing the burden of caregivers

Is the work clearly and accurately presented and does it cite the current literature?

Yes

If applicable, is the statistical analysis and its interpretation appropriate?

Yes

Are all the source data underlying the results available to ensure full reproducibility?

Yes

Is the study design appropriate and is the work technically sound?

Yes

Are the conclusions drawn adequately supported by the results?

Yes

Are sufficient details of methods and analysis provided to allow replication by others?

Yes

Reviewer Expertise:

Dementia

I confirm that I have read this submission and believe that I have an appropriate level of expertise to confirm that it is of an acceptable scientific standard, however I have significant reservations, as outlined above.

## References

[1] : Cerebrospinal Fluid Aβ42 Levels: When Physiological Become Pathological State. *CNS Neurosci Ther* .2015;21(12) : 10.1111/cns.12476 921-5 10.1111/cns.12476 26555572PMC6493161

[2] : CSF biomarkers for Alzheimer's Disease: levels of beta-amyloid, tau, phosphorylated tau relate to clinical symptoms and survival. *Dement Geriatr Cogn Disord* .2006;21(3) : 10.1159/000090631 131-8 10.1159/000090631 16391474

[3] : Frailty among Alzheimer's disease patients. *CNS Neurol Disord Drug Targets* .2013;12(4) : 10.2174/1871527311312040010 507-11 10.2174/1871527311312040010 23574166

# Reviewer report: Reviewer response for version 1

**DOI:** 10.21956/hrbopenres.14809.r34689

**Published:** 2023-06-29

**Authors:** Jennifer Bray

**Affiliations:** 1 University of Worcester, Worcester, England, UK

There were a few areas where I felt the work could have been presented more clearly or more detail could have been provided to help the reader better understand what was done. Apart from a possible lack of detail at times, I did not have any particular issues with the study design. As per my comments below, more detail could be provided to make the methods and analysis clearer. At present I’m not sure it would be possible to replicate the work. More detail would help the reader to fully interpret the findings and provide context, such as the number of people who responded to each theme. The source data can be requested but is not automatically provided in the article. The conclusions are clearly based on the results, but the points raised in my comments make it tricky to appreciate the full context.

 It may be that everything is fine and it’s just how it’s been presented that raises a few queries for me, but in its current format the level of detail and lack of consistency in some areas mean that it’s difficult to have full confidence in the findings. I think the findings are interesting and important, but just that extra bit of work to strengthen the article would give them more weight.

**Comments to consider and address:**

- I would suggest being aware of the language used to avoid automatically being negative. For example, in the abstract at the end you talk about ‘conduct research to address the burden of dementia’. Please find an alternative way to express the point, such as ‘conduct dementia-focused research’. If you are talking about the economic burden on the healthcare system (as suggested in your main introduction) please make this clear. At present it reads as a personal/emotional burden on families. I’m not convinced by the use of ‘burden’ in your introduction either.
- In your introduction you talk about prevalence rates. I would expect to see this expressed as a % or so many people per 10,000 or 100,000 but you’ve just given the overall number of people with dementia in Ireland. You may have got those numbers by using the prevalence rates, but for me ‘between 39,272 and 55,266’ are not prevalence rates in their own right.
- When describing the anonymous survey, is it possible to give an indication the types of basic demographic information collected, and why it was needed?
- On page 5 you repeat a sentence about recruitment. In the first column when talking about the workshops you say how participants were recruited. In the second column when talking about the workshop participants you say how they were recruited. I would suggest the first instance could be removed as it makes more sense in the second instance.
- There is also some repetition in terms of saying how many people were involved in each workshop (on page 5 first column and page 6 first column). It feels like you could just say it in one place.
- Analysis – apologies if I’ve missed this, but it would be useful to have more information about the scoring of the workshops. It might just be a case of clarifying how many questions were within each of the six over-arching themes, because on seeing Table 5 I was confused about how one of the research domains could have 31 votes from 13 participants. It might sound silly, but it shouldn’t be up to the reader to try and work it out for themselves by looking at Table 7, especially when the research domains/over-arching themes have different titles for the survey and the workshops.
- Demographics for survey respondents – a lot of the information given on page 17 is what I would expect to find here. At the moment it’s basically 108 people took part and have a look at Table 2. It makes it feel like collecting the demographic information wasn’t worth doing as you pretty much ignore it. If you moved the actual information from page 17 to here it would make more sense, then in your page 17 discussion you could indicate how your participants compared to the global survey. E.g. ‘The majority of professionals in both the Irish and global surveys identified themselves as researchers or clinicians at 75% (n=108) and 88% (n=142) respectively’. This might be a bit simplistic, but presenting your demographic results for the first time in the discussion just feels odd. Also, it would be good to have something in the text about the other demographics that you captured, rather than just leaving it to the reader to look at Table 2. I’m not saying you need to do any deep analysis, but one short paragraph to give an overview of who the professionals were is all it would need.
- Top 10 research priorities – it is probably worth clarifying that this is for professionals. Also, based on the research domain titles I’d suggest that ‘focused on the quality of care and delivery of services’ is more accurate. Using the full domain title in Table 3 would also be better (and more consistent with Table 4) rather than just ‘quality of care’. I’d suggest listing the other two themes in the reverse order as ‘timely and accurate diagnosis’ was ranked lower than ‘diversifying therapeutic approaches’ but you’ve listed it first which implies the opposite. Similarly, when listing the four priority areas within quality of care, I’d put them in the order they were ranked to give a more accurate picture. (If they are in order in the text, you need to change Table 3). Is there a reason why the fifth priority area isn’t listed?
- Top 20 research priorities – I initially found this confusing as it seemed very similar to the top 10, especially as you talk about eight of them in particular. It might be slightly clearer if you say something like ‘included a total of eight research avenues’. When talking about ‘clinical-translational research’ it might be worth using the full title given in Table 3 to help the reader tie everything together. It might also be helpful to reorder the start of the paragraph to make it read better. For example, something like ‘In addition to ‘diversify…trials’ which was ranked in the top ten, the other research avenues relating to the theme of ‘clinical-translational research (full title)’ in the top 20 priorities…’
- I think it would be beneficial to have some consistency. You talk about ‘Research domains’ in Table 3 but in the text you talk mainly about over-arching themes. On page 8, column 2 you even switch between the two terms within the same sentence.
- Table 6 – It might be useful to say how much the money allocated is out of. I know you had 7 carers and they had EUR 5,000 each, so theoretically it should be out of EUR 35,000, but reminding the reader of this would help to provide context. It’s interesting, and a bit confusing, therefore that only EUR 25,000 appears to have been allocated. The reason for this disparity should be explained (e.g. if only 5 carers used their money, say it). Listing all six research domains and showing that the final two got no money allocated would also be beneficial to give the full picture.
- Page 17, both columns – do you actually need the two paragraphs about ‘Sex or gender’ as you basically already said about this in the methods section? I know the professionals one is about analysis rather than collecting the information in the first place (which makes me wonder why you bothered to ask about their gender if you weren’t going to do anything with the info), but still.
- Page 18, column 1 – is there a better way to express what you’re saying in the first paragraph that maybe doesn’t involve giving the EUR values as you’ve only just given those values in the paragraph at the end of page 17. Maybe focus on better drugs and treatment being the highest priority for workshop participants, but was second highest in the money game carried out with carers. It just feels a bit repetitive at the moment even though you are actually saying something different in the two paragraphs.
- Page 18, column 2 – when you talk about completion rates, it would be useful to provide more clarification what you mean. The way it reads at the moment suggests that although 108 people responded to the survey, some themes only had 41 people responding, with at most 62 people responding to other themes. This doesn’t seem like a great response rate, and feels like the sort of thing that should be mentioned in your results. Maybe when presenting the rankings or overall scores in the tables there should be some indication of how many responses they are based on. It would be easy for the reader to assume that something was ranked 1 ^st^ across all 108 people, so if it’s actually just based on 50 people that presents a different picture.
- Also, you say that respondent numbers decreased as the survey progressed, so it would be interesting to know the order in which the research domains were presented as this could have an impact. For example, were the themes that people ranked highly to ones earlier in the survey? Could some themes not be considered a priority because people didn’t actually get that far through the survey, or were fed up by that point and not paying enough attention?

​​​ **Minor points**

- Introduction – should it be ‘represent a significant’ rather than ‘represents a significant’?
- Introduction – should there be a ‘ – ‘ after ‘both emotional and economic’ rather than a ‘,’?
- Introduction, column 2 – you’ve already set up the abbreviation HRB in column 1, so why do you not use it at the top of column 2? Also, I’m confused by the use of HSE as it looks like it’s set up as an (odd) abbreviation of National Dementia Office twice within one paragraph. If it’s actually a different abbreviation it needs to be expanded the first time it’s used.
- Introduction, column 2 – is it ‘steering group’ or ‘Steering Group’ as you seem to use both?
- Various places – when you use / do you have spaces around it or not? At the end of introduction column 2 you use ‘and/or’ and ‘dementia/ dementia’ in the same sentence. Please be consistent.
- Page 5, column 1 – in your list of criteria why do ii and iii not have ‘?’ at the end?
- Page 6, column 2 – in your second paragraph about ethics I would suggest saying that ‘Ethics approval was not required for the workshops’ rather than ‘not obtained’ as the current wording immediately made me think you’d done something wrong and unethical. Also, you’ve already set up the DRAT and DCCN abbreviations on page 5, column 2, so you don’t need to do it again here, just use them.
- Top 4 research avenues – it feels odd to give a range of scores with the higher score first. I would expect to see ‘from 0.57 to 0.89’ but I’m willing to be overruled on this. (although having seen later on page 17 that you say ‘from 0.81 to 0.89’ it feels like you should be consistent in terms of whether you give the higher or lower score first).
- It also seems odd to jump from Table 4 to Table 7. I can see why it’s done based on where it was possible to position the tables, but it just makes me as a reader wonder what happened to tables 5&6.
- I’m not sure you need to keep reminding the reader that the workshop participants were ‘(people with dementia and carers)’, especially as the first time you do this is just after the title ‘Priority ranking by people living with dementia and carers’.
- Page 17, column 1 – when saying ‘In link with findings from this study and the global survey’ it might be useful to make it clear that ‘this study’ relates to the PSP study not the work you’ve done. It probably wouldn’t be an issue normally, but the placement of Table 7 breaks the flow for the reader making it trickier to keep track of what you are talking about. Similarly, when saying ‘Comparing these findings to the Irish survey’ it might be better to say ‘Comparing these PSP findings to the Irish survey’ or however you want to refer to the PSP study – you say ‘the UK’s PSP exercise’ later on, so maybe choose a consistent phrase and stick with that throughout this section.
- Page 19, column 1 – why are you setting up the DRNI and ASI abbreviations here (again), when you’ve been using them throughout the article?

Is the work clearly and accurately presented and does it cite the current literature?

Partly

If applicable, is the statistical analysis and its interpretation appropriate?

Partly

Are all the source data underlying the results available to ensure full reproducibility?

No

Is the study design appropriate and is the work technically sound?

Yes

Are the conclusions drawn adequately supported by the results?

Yes

Are sufficient details of methods and analysis provided to allow replication by others?

Partly

Reviewer Expertise:

Dementia

I confirm that I have read this submission and believe that I have an appropriate level of expertise to confirm that it is of an acceptable scientific standard, however I have significant reservations, as outlined above.
